# A Political Picnic

**Published:** 1882-01

**Authors:** Thos. K. Beecher, H. H. Rockwell, C. A. Collin, E. W. Krackowizer


### A POLITICAL PIC-NIC.

I am asked to preface the three papers that follow by a few words of my own.

The reader will find them to be highly intelligible and profitable. By each he will learn what are the throbbing questions of social welfare that are forcing themselves upon public attention:- -Money, labor, land, debt, credit, usury or interest. The full and fair discussion of these topics will prove to be indeed a popular education.

Besides these, the question of revenue, involving tariffs, excises, and direct taxes, should have place and discussion.

It is certain that *all* these questions will not be—cannot be—discussed and settled at once. Upon which one the ten million eyes of voters will first focus, cannot now be predicted.

At date, the political ocean after its high-tide of 1862–4, is at "low-water slack." Questions bob up and down upon the water restlessly like the chips and rubbish in the docks and little bays of our coasts. By and by, we shall begin to feel a tide,—a movement all along shore.

During this present time of "slack" and of stationary and sloppy restlessness, our elections will be decided not by discussions of policy, but by thoroughness of organization, and the size of the "campaign funds."

The party in power can usually perfect the best organization, and draw upon the largest fund. The party in power naturally grows conservative. The man on top is ever contented with the heap as it is.

The Republican is the conservative party to-day, as the Democratic party once was. The National is the party of free thought, idealism and clamor, as the abolition or anti-slavery party once was. The Democratic is the party in expectancy like the old Whig party. But unlike that party of genteel fossils, it seems highly probable that it will learn the inevitable from the Nationals; and when a half dozen of its great leaders are gathered to their fathers, the young democrats will once more voice the demands of the whole people; demands that are never heard by the ears of your contented conservative, nor, with his consent, conceded.

—— I ask attention to the following papers, and in the name of many readers express beforehand our gratitude to the editor and proprietor of the BISTOURY for their publication. May his salutary example be followed by other newspapers, and thus give their readers a chance to "prove all things and hold fast that which is good." Read, ponder, decide, act.

THOS. K. BEECHER.

---

A wise judgment as to what the Democratic party will assume or teach should be based on its past record and present attitude, which may be gathered from the acts and utterances of its leaders and organs, and from its platforms, which embody the consensus of party doctrine as held at the time of their adoption.

I find nothing in these "as to the duty of government in the forcible collection of debt"—a subject which has not been made a political issue, and about which I am therefore unable to state. For convenient treatment I will restate the other themes submitted to me, thus:

1. Money and the public debt.
2. Public lands.
3. Convict labor.

The record since 1868, the date of the first national convention after the war, which marks an epoch in our history, will afford ample data for our conclusions.

I. The platform of 1868 demanded "payment of the public debt as rapidly as practicable, * * * and where the obligations of the government do not expressly state on their face that they shall be paid in coin, they ought in right and justice to be paid in lawful money of the United States." (Greenbacks.)

That of 1872 demands "the payment of the interest on the public debt and a moderate reduction annually in the principal thereof"; affirms that "the public credit must be sacredly maintained"; denounces "repudiation in every form and guise"; and demands "a speedy return to specie payments." That of 1876 denounces "the failure to make good the promise of the legal tender notes, and to accumulate a reserve for their redemption," and demands "a system of wise finance which shall enable the nation to assure the whole world of its ability and readiness to meet any of its promises at the demand of the creditor entitled to payment." That of 1880 "pledges the party anew to the constitutional doctrines embodied in the platform of the last national convention," and to "honest money, consisting of gold and silver, and paper convertible into coin on demand, and maintenance of the public faith, State and national."

The campaign of last year afforded an unusual amount of political discussion on the stump and in the press, which continued all through the ensuing session of Congress, and until after the passage and veto of the refunding act; and I believe that without exception the party leaders, speakers and writers were in accord with the platform on these subjects. As to what the party has done with reference to them, my space will permit but a brief glance. Since 1860 it has been in power in the Senate but two years, and in the House of Representatives but six. Without attempting to review the legislation of those years, it is safe to assume that its acts have been in accord with its avowed principles. The remonetization of silver was a democratic measure. The retrenchment of public expenditures and application of the monies thus saved in payment of the public debt, was a democratic accomplishment in the face of strong republican opposition.

The three per cent. funding bill was a democratic measure, vetoed by a republican President at the demand of the national banks. The aim and efforts of the democratic party have been directed to the protection of the people from legislation in the interest of bondholders and capitalists, who want to make money scarce and dear, so that it may bring high rates of interest, and enhance the value of bonds and other evidences of indebtedness, as compared with all other kinds of property.

To this end it opposed the policy of forced resumption, without adequate means being provided for its accomplishment. But for the remonetization of silver, the wide-spread ruin which that policy entailed upon the debtor class would have been still more disastrous. We may safely assume that the democratic party will continue to oppose a policy which shall favor the creditor at the expense of the debtor class, the rich at the expense of the poor, the strong at the expense of the weak. That its interpretation of "gold, silver and paper convertible into coin," will include the free coinage of silver, and a sufficient amount of greenback currency for all business requirements. That the government shall issue all money without the intervention of the national banks, which have shown their power and willingness to use their special privileges as a menace to business interests, for the purpose of coercing Congress into compliance with their wishes. That in the management of the bonded debt it will insist that all efforts shall be directed to its bearing the lowest rate of interest compatible with its early extinguishment.

II. Commencing with 1868 and down to 1880, every democratic platform has contained an emphatic declaration in favor of the distribution of the public lands among actual settlers. "The public lands should be distributed as widely as possible among the people, either under the preemption of homestead lands, or sold in reasonable quantities to none but actual occupants, at the minimum price established by the government." 1868.

"The public lands should be held sacred to actual settlers." 1872. "Reform is necessary to put a stop to the profligate waste of the public lands, and their diversion from actual settlers by the party in power, which has squandered 200,000,000 of acres upon railroads alone, and out of more than thrice that aggregate, has disposed of less than a sixth to tillers of the soil." 1876. "Public lands for actual settlers," 1880. During the time that the party was in power in Congress, as above stated, it did not vote an acre of land to any but actual settlers

The homestead law, so much vaunted as a republican measure, was first introduced into Congress twenty-seven years ago by a democratic member from Tennessee, named Andrew Johnson.

III. The management of convict labor is one of State, and not national policy. In 1868 a democratic legislature appointed a committee to investigate the subject and make suitable recommendations. It reported in 1869 that the convict system was wrong in principle, and recommended State employment under suitable restrictions as to numbers employed at any one trade, and the sale of the products at not less than market prices. A democratic governor, John T. Hoffman, in his message of that year made similar recommendations. In 1870 the democratic legislature passed the act to establish the State Reformatory, which provided that "the contract system shall not be permitted therein." In 1871, "Our Member," Mr. Hill, introduced and caused to be passed in the Assembly an act to abolish the contract system of prison labor, and provide for State employment under the restrictions above mentioned, but the contractors rallied and defeated it in the Senate. Since then the democratic party has not had a majority in the Senate, and but once in the Assembly, (1875) and has consequently been powerless to legislate on the subject, which has not been discussed or legislated upon until the republican legislature of last winter removed the prohibition of the act of 1860, and applied the contract system to the State Reformatory.

From the above, it is fair to infer that the democratic party will favor neither the contract system nor the support of convicts in idleness at the expense of the taxpayers; but will insist that the State shall so employ them and dispose of the products of their labor as not to injure honest industry by unfair competition.

My final conclusions are that the democratic party will assume and teach that all money shall be issued by the government, and consist of gold, silver and greenbacks; that the bonded debt shall bear the lowest rate of interest compatible with its constant reduction by payment and its early extinguishment; that the public lands be held sacred to actual tillers of the soil; and that convict labor ought not to be let by contract, but should be managed and employed by the State so as to inflict the least possible injury upon honest labor.

H. H ROCKWELL.

---

I have been requested to make a statement of the probable doctrine and tendency of the republican party upon certain topics in the field of political economy.

The doctrines of a party are presumptively to be obtained from its platforms. But the imposing platitudes and vague generalities of party platforms are sometimes purposely misleading, and often furnish but a barren and unsatisfactory field for the study of the spirit and tendency of a party. From a study of party platforms, we will always find all parties in favor of an honest and economical administration of the government, opposed to monopolies, in favor of such an adjustment of the relations of capital and labor that each shall receive the fair reward to which it is justly entitled, and other equally safe and equally meaningless propositions. There is in fact a far greater difference in general spirit and tendency, between the two leading parties at the present time than their platforms indicate.

The republican party was founded by men of radical principles. The accession of the party to power in 1860 was a triumph of the radical and revolutionary elements of society. Twenty years uninterrupted control of the national government, however, has transformed the republican party into preeminently the conservative party. A wholly new generation of leaders are now directing its policy, a wholly new and diverse set of sentiments now impel its supporters. A party that has had so long a lease of power must by the very fact of justifying its own ways, become an advocate of the old ways; by justifying its own policy it becomes an advocate of the present order of things. A party with snch a past must, of necessity, have become eminently orthodox, respectable, and conservative.

The republican party, therefore, justly has the support of the vast majority of those whose interests or dispositions lead them to prefer stability, order and permanence; of those who have laid their plans upon the basis of the continuancc, at least in its essential features, of the financial system under which the wealth of modern times has been accumulated and distributed. As naturally and properly, there will be found outside of the republican party, the over fastidious and critical who can support no system which they do not find absolutely perfect, but who lack the energy to attempt its improvement, the impracticables or "incompatibles," as Matthew Arnold terms them, as well as the fanatics and reformers who propose to revolutionize—to tear down and build over—every system in which they can find any fault, and likewise the considerable company of Adullamites, made up of "every one that is in distress, every one that is in debt, and every one that is discontented."

The democratic party has undergone an equally complete reversal of its general spirit. It has been so long trying to catch on to the popular favor with one new scheme after another, that it has become restless, uncertain and discontented, "tossed to and fro and carried about with every wind of doctrine," losing confidence in itself and its principles, an habitual seeker after some change to be wrought; while the radical and revolutionary reformers are forming the dead in earnest, unorthodox and despised, but by no means despiscable, greenback party.

This general conservative tendency of the republican party is the leading feature of its position upon almost every one of the topics proposed.

1st. *As to money, its manufacture, issue, and as an article of merchandise.*

What are known as hard money doctrines are orthodox, in the sense of having the support of the great majority of the most learned authorities, and of the more wealthy and educated people. The centre of gravity of the republican party is farther to the eastward than its centre of magnitude, and the easterly portion of the republican party are substantially unanimous in a firm advocacy of hard money, while the few western soft money republicans are timid and embarrassed. There is a strong tendency in the republican party to oppose even a limited coin age of silver, and it is the only party at all likely to insist upon the coinage of gold only; but bimetallism—the unlimited coinage of gold and a limited coinage of silver—will probably give a sufficiently hard money to satisfy and unite the party. As the greenbacks now in circulation have for a long time and will probably always hereafter be practically at par with coin, there is no objection on the part of hard money men to their continued circulation for an indefinite time. But the republican party will not allow the possibility of any further issues of greenbacks to endanger, as they believe it would, the permanence of the present coin value of the greenback dollar.

As to bank currency, the republican party proudly claims its responsibility for the present national bank system. But the party will probably make no objection to any practicable system of free banking, which absolutely secures the redeemability of the bank currency in coin at par value. They will never allow a bank currency again to be issued which fluctuates in value with the credit of the bank, becoming worthless when the bank breaks. Thus keeping the entire paper currency at par with coin, they will place no restrictions upon dealing with money as with any other commodity, with the one exception of maintaining reasonable usury laws.

2d. *The duty of government in the forcible collection of debt.*

Reasonable exemption laws will of course be maintained, and probably a bankrupt law will be re-enacted. But any proposition to directly abolish any of the present laws for the collection of debts, even though it might not be so revolutionary in its practical workings as it looks, still looks altogether too revolutionary to receive any consideration whatever from the republican party.

3d. *The treatment of the public bonded debt.*

The republican party will continue its present policy of paying the bonds in full in gold, and keeping them exempt from taxation until paid, in accordance with the spirit and letter of the contract as they understand it; and in the meantime keeping the rate of interest thereon as low as possible.

4th. *Convict labor and its management.*

This is as yet an unsolved problem. The present system is admitted on all hands to be exceedingly faulty and unsatisfactory. Any practicable plan which will remedy or ameliorate the evils of the present system, without involving new and greater evils, will be eagerly adopted by the republican party. They will strive, however, to adopt some more humane and economical system than to let the convicts "rot in idleness," the only definite remedy yet proposed by democratic statesmanship, outside of wise looking generalities, and they will also hesitate before adopting the proposal of our local greenback leaders to send our convicts to some lonely isle, and there leave them to kill each other off.

5th. *Public lands and their sale.*

Land limitation laws are obnoxious to all the traditions of our modern financial system. The republican party is in favor of disposing of the public lands in small farms to actual settlers, but it will not interfere to prevent any man from buying all the lands of the actual settlers that he can pay for.

There will probably be no further grants of public lands to railroads. The trans-continental railway lines to which grants have chiefly been made, were essential to the national unity, and could not at the time have been built by private enterprise. That method of cementing the Union was more economical and effective than war. Those necessities are now fully met, and future railroad corporations can be safely left to their own resources. Moreover, the abuses that have been connected with grants of public lands to railroads, and for which no political party could be held responsible, have rendered unpopular the entire system of government aid to corporate enterprises which contribute to the public welfare, but appear unprofitable to the corporations, a system which the republican party has heretofore been inclined to favor.

C. A. COLLIN.

---

Happily the NATIONAL party's champion need employ little more than shears and paste to present his views upon the vital questions propounded—so full, fair and frequent have been its declarations as to all but one of them. Accordingly I shall venture to interject no more than brief, running comment between the extracts from our National, State and County Platforms and committee addresses—abiding in the hope that upon some future occasion the scope of this symposium may be extended to include a full discussion of the principles now but enunciated.

Permit me, then, to premise that our late State and County Conventions endorsed and reasserted the principles of our last National Platform in general as well as specific terms.

I. *Money—its manufacture, issue and intrinsic value.*

Article one of our National Platform reads:

The right to make and issue money is a sovereign power to be maintained by the people for the common benefit. The delegation of this right to corporations is a surrender of the central attribute of sovereignty, void of constitutional sanction, conferring upon a subordinate irresponsible power, absolute dominion over industry and commerce. All money, whether metallic or paper, should be issued and its volume controlled by the government, and not by or through the banking corporations, and when so issued, should be a full legal tender for all debts, public and private.

By way of explanation our State Committee adds:

The NATIONAL Party holds that the right to make and issue money is a sovereign power which belongs to the general government only, and that that power, under the federal constitution, cannot be delegated to any state, corporation or individal. That the general government can through its laws place its stamp on any material and make the material so stamped, the lawful money of the country, by making it a *full legal tender in paymeut of all private debts; and receivable for all public dues.* It holds that the volume of money should be equal to the business requirements of the country, so that the people will not be compelled to pay interest on seven dollars, for every dollar in circulation as they do now. It holds that gold and silver are utterly inadequate in their amount to furnish the supply of money required, and that the only purpose *metal* money now serves in this country is to promote the interests of usurers and professional money-lenders, and that its use as money will soon be regarded a relic of barbarism. It holds that the value attached to all money is governed by the confidence in which it is held by its owner, and the American people have so much confidence in the stability and perpetuity of their government, that they will always accept the money it issues because they have that confidence. It holds that when money has been issued in sufficient amount to meet the demands of business, its volume should be made permanent; so as to give permanence to the value of property, and the employment and compensation of labor.

Now, all this reduced to plain, homely speech signifies: a chunk of gold, silver or nickel is not money; no more is a strip of paper. It is the stamp upon either that makes them such. Furthermore, the right to coin money *i. e.*, to impress the money-stamp upon *any* material inheres in Congress. [Examples:—In intrinsic value, the trade dollar exceeds the Bland dollar by 7½ grains silver fine,—yet in the eye of the law it is but bullion which can not legally satisfy any indebtedness. Similarly the National Bank Note is not a legal tender, whereas the Greenback is.] Hence all *money* is "*fiat*" money, and it goes without saying that nothing should by the government be permitted to circulate as money, except it be a full legal tender; nor ought this right ever to be delegated—in part or entire. And finally, in so far as substances having intrinsic value are chosen as the material for money—the same, *i. e.* "commodity" money, is of evil; for *qua* commodity it does not possess those qualities of flux and elasticity without which money cannot subserve its true uses, viz: Serve as a measure of value and medium of exchange, that and that alone. Hence we claim that the Government should issue only "*absolute*" *money—i. e.* tokens of its indebtness, constituted by its fiat, the law—a legal tender for the settlement of private indebtedness as well; and returnable to the treasury in payment of taxes, costum dues, &c., *i. e.* "*a full legal tender in payment of all debts, public and private.*"

II. *Debt—the Government's duty as to its forcible collection.*

The NATIONAL Party has not *formulated* any theories or demands in regard to this question, but if my individual judgment be not at fault —and the drift of the intelligent dominating opinion be indeed, as I take it, in favor of boldly supplementing the slogan: "Anti-Monopoly—Absolute Money" with its corrolaries:" "Free land, no rent—free money, no usury,' then its position is simply this: idle capital is, or rather should be unproductive; hence no one ought to lend of his abundance to another upon usury, but should gladly risk loss and share profit, (whichever the venture may yield) with his partner the debtor. Hence, since it oppresses the honest unfortunate and fails to eliminate the dishonest trickster, a forcible collection of debt under the law is evil.

III. *The public Bonded Indebtedness—the Government's treatment of and policy as to the same.*

Article two of our National Platform reads:

The bonds of the United States should not be refunded, but paid as rapidly as is practicable, according to contract. To enable the government to meet these obligations, legal tender currency should be substituted for the notes of the national banks, the national banking system abolished, and the unlimited coinage of silver as well as gold established by law.

Simple honesty, and politic frankness, as well, demand that it be acknowledged: the final clause of this paragraph should be looked upon as a sop thrown to the hard money Cerberus; a piece of misguided policy and trimming which would not to-day be considered necessary. To perpetuate our National debt for no other conceivable purpose than to subsidize the "National Banking Monopoly," were criminal folly indeed. For the major part of twenty years these institutions have not only drawn six per cent. interest *untaxed* upon their original investment, so lodged that neither moth may eat nor rust corrupt; but they have in addition by the loan of their I. O. U's (*i. e.* their circulation) been able to "shave" from six per cent. to at least twelve per cent. more. All this immense profit accruing to a class of monopolists who depreciated the Greenback by craftily insisting upon that fatal proviso as to its functions, viz: "Receivable for all debts, public and private, *ex-*

*cept custom dues and interest on Public Bonds,"* thereby enabling themselves to purchase these Public Bonds at an average rate of fifty cents on the dollar, and thus compelling the Government to issue twice as great an amount as would otherwise have been necessary; accruing to a class of monopolists, who, besides thus increasing their capital two hundred per cent., managed to "strengthen the public credit" by substituting the word "Coin" for "lawful money" when they had succeeded in demonetizing silver; and who, when silver had been again remonetized, made "coin" mean "gold." Hence we NATIONALS say pay off these bonds as fast as the Treasury surplus will permit, strictly and honestly according to the letter of the contract; thereby, ridding ourselves of debt and banks at one blow and of all that immense drain of interest, which their existence entails.

IV. *Convict Labor—and its management.*

Article three of our National Platform reads:

Labor should be so protected by National and State authority as to equalize its burdens and insure a just distribution of its results; the eight hour law of Congress should be enforced; the sanitary condition of industrial establishments placed under rigid control; *the competition of contract convict labor abolished;* a bureau of labor statistics established; factories, mines and workshops inspected: the employment of children under fourteen years of age forbidden, and wages paid in cash.

At our late County Convention it was further

*Resolved*, That we are opposed to any system whereby prison labor is made to compete with honest toil, and that our candidates for Senate and Assembly shall pledge themselves to this principle.

Upon the strength of which declaration the following petition was circulated by us and largely signed throughout the county.

"We, the undersigned, irrespective of party pledge ourselves not to vote for any candidate for Senator or Assembly, who will not pledge himself to oppose all measures favoring Contract Convict Labor."

As the competition of negro slavery in the South of other days bred "white trash," of low-lived, poverty-stricken, sodden, shiftless ignorance—so the merciless competition of contract convict labor at seven cents per hour per head, must sooner or later degrade, impoverish and brutalize honest labor all over the land—such as it is brought into direct competion with, to say the least. For the honest mechanic cannot compete with this cheap—nay this *dirt* cheap, immoral and impersonal laborer, while providing shelter, clothing, food and schooling for him besides. For if on a dollar *per diem* he succeed in accomplishing this, he must of necessity fail of satisfying his own wants not to mention those of his family. So our Platform says elsewhere: "as negro-slavery was cheap labor, so cheap labor is but slavery."

V. *Public lands—their pre-emption and sale.*

Article eleven of our State Platform reads:

We protest against the proposed sale of public lands in Minnesota, as advertised by the Secretaries of the Interior and Treasury, except to actual settlers, and in parcels not exceeding 160 acres. And by way of explanation our StateCommittee adds: It holds that the public lands belonging to the Government should be held for actual settlers and not be granted or sold in large tracts to corporations or individuals.

For unto the great transcontinental railway monopolies were given out-right by way of gratuitous subsidy alternate sections of the richest mining and farming lands in the original pnblic domain—a tract as large as the New England and Middle States, plus Ohio, Illinois and Iowa—with millions upon millions of Government bonds thrown in, [$64,623,512.00+36,-104,186.81 interest paid,] the latter "secured" by a worthless *second* mortgage. But when the late Hendricks B. Wright, sought to introduce into Congress his bill providing for the ready pre-emption of small tracts of public lands by poor laboring men, the Government to lend these actual hard working settlers from $500 to $1,000 at one per cent. upon a first mortgage—he was not allowed to do so and hooted at all over the country as an arrant lunatic, &c.

And finally article fourteen of the National Platform reads:

In the furtherance of these ends we ask the co-operation of all fair-minded people. We have no quarrel with individuals, wage no war upon classes, but only against vicious institutions. We are not content to endure further discipline from our present actual rulers, who having dominion over money, over transportation, over land, labor, and largely over the press and machinery of government, wield unwarrantable power over our institutions, and over life and property.

All of which is respectfully submitted by

E. W. KRACKOWIZER.

---

SUBSCRIPTIONS ARE DUE.—Do not delay sending yonr fifty cents for another year, but send at once.

